# Management of Direct Oral Anticoagulants in Patients with Atrial Fibrillation Undergoing Cardioversion

**DOI:** 10.3390/medicina55100660

**Published:** 2019-09-30

**Authors:** Giuseppe Coppola, Girolamo Manno, Antonino Mignano, Mirko Luparelli, Antonino Zarcone, Giuseppina Novo, Egle Corrado

**Affiliations:** Division of Cardiology, University Hospital “P. Giaccone”, University of Palermo, Via del Vespro 129, p.c. 90127 Palermo, Italy; giuseppe.coppola@policlinico.pa.it (G.C.); girolamomanno@hotmail.it (G.M.); mirkolupini@gmail.com (M.L.); zarconeantonino.91@gmail.com (A.Z.); eglecorrado@gmail.com (E.C.)

**Keywords:** direct oral anticoagulants (DOACs), atrial fibrillation (AF), electrical cardioversion (EC)

## Abstract

Atrial fibrillation the most common cardiac arrhythmia. Its incidence rises steadily with each decade, becoming a real “epidemic phenomenon”. Cardioversion is defined as a rhythm control strategy which, if successful, restores normal sinus rhythm. This, whether obtained with synchronized shock or with drugs, involves a periprocedural risk of stroke and systemic embolism which is reduced by adequate anticoagulant therapy in the weeks before or by the exclusion of left atrial thrombi. Direct oral anticoagulants are safe, manageable, and provide rapid onset of oral anticoagulation; they are an important alternative to heparin/warfarin from all points of view, with a considerable reduction in bleedings and increase in the safety and quality of life of patients.

## 1. Introduction

Atrial fibrillation (AF) is the most common cardiac arrhythmia worldwide. It is nowadays a real “epidemic phenomenon” considering an incidence of approximately 25% in patients aged >40 years with high prevalence in elderly patients [1,2,3,4,5,6]. The worldwide prevalence of atrial fibrillation in the near future will necessitate mandatory, safe, and effective management [5,6].

**Figure 1:**
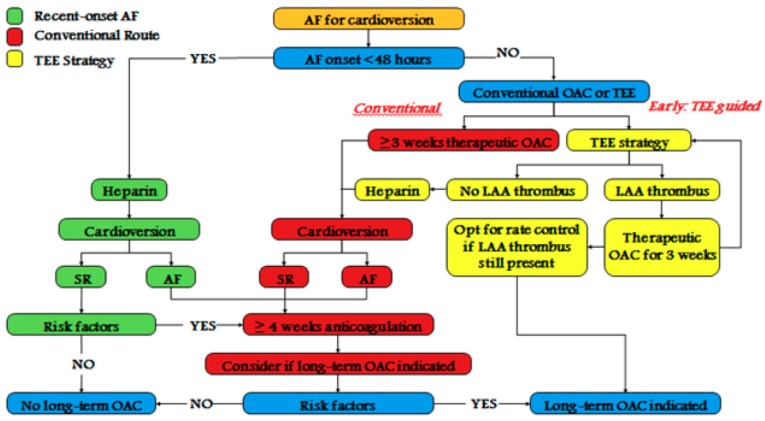
Suggested flow chart for atrial fibrillation (AF) cardioversion on the basis of the current European Society of Cardiology (ESC) Guidelines. Transesophageal echocardiogram (TEE), anticoagulant therapy (OAT), left atrial appendage (LAA), sinus rhythm (SR).

Cardioversion is defined as a rhythm-control strategy that, if successful, restores normal sinus rhythm. There are two types of cardioversion: pharmacological (the preferred strategy in patients presenting with recent-onset AF; within 48 h) and electrical (the preferred strategy when AF is prolonged). Cardioversion is very important in the management of AF [7,8]; indeed, delays in cardioversion promote atrial remodeling and difficult sinus rhythm restoration, increasing the likelihood of postcardioversion AF recurrence and adding further thromboembolic risk [8,9,10]. In fact, sinus rhythm restoration, either obtained with electrical cardioversion or with drugs, carries a periprocedural risk of stroke and systemic embolism which is decreased by adequate anticoagulation in the weeks before cardioversion or excluding left atrial thrombi before the procedure [1,9,10,11,12,13,14] (see Figure 1). For these reasons, prophylactic anticoagulation represents a cornerstone of peri-cardioversion management in patients with AF [1,2,3,4,5,6,7,8,9,10,11,12], even if, in patients with datable AF (less than 48 h), it is usual to perform cardioversion without transesophageal echocardiogram (TEE) or antecedent oral anticoagulant therapy (OAT) [12,13,14]. Randomized controlled trials (RCTs) comparing direct oral anticoagulant (DOAC) therapies in patients with AF duration of <48 h are not available. The same applies to patients with hemodynamic instability and AF that can undergo cardioversion immediately [12]. Long-term OAT after cardioversion should be based on the long-term risk of stroke using the CHA2DS2-VASc (Congestive Heart failure, hypertension, Age ≥75 – doubled-, Diabetes, Stroke –doubled-, Vascular disease, Age 65–74, and Sex female) risk score. If the duration of AF lasted more than 48 h, or its onset is not evaluable, the periprocedural risk of thromboembolism can be as high as 5–7% without anticoagulant therapy [12,14]. In this clinical situation, current guidelines recommend therapeutic anticoagulation for at least 3 weeks before and at least 4 weeks after cardioversion [12,13,14] (see Figure 1). It is important to underline that the highest risk of thromboembolism is within the first 7 days after cardioversion (>80% of events) with the greatest risk within the first 72 h [15]. An embolic event after cardioversion can be due both to the fact of left atrial thrombi migration or to the subsequent formation and migration of de novo thrombi caused by postcardioversion atrial stunning [8]. The single biggest risk factor for thrombus formation is inadequate anticoagulation [1,12,13,14,15,16]. In the current European Society of Cardiology (ESC) Guidelines for the management of AF [12], the recommendation for anticoagulation with warfarin before cardioversion is in first class for a time ≥3 weeks and must be continued for ≥4 weeks after the procedure, based on pathophysiological and observational data [12]. Compared to vitamin K antagonist (VKA) therapy, the use of DOACs offers potential advantages in the setting of cardioversion, although their use is recommended in Class IIa in the last ESC Guidelines [12]. These advantages include a faster onset of therapeutic anticoagulant effects, avoidance of heparin bridging, and improved quality of life avoiding the mandatory blood sample to control international normalized ratio (INR) range when vitamin K antagonist are used [14]. 

The use of DOACs in this setting of patients is based on subgroup analyses of Randomized Evaluation of Long-Term Anticoagulation Therapy trial (RE-LY) for dabigatran [16], from Rivaroxaban Once Daily Oral Direct Factor Xa Inhibition Compared with Vitamin K Antagonism for Prevention of Stroke and Embolism Trial in Atrial Fibrillation trial (ROCKET AF) for rivaroxaban [17], and from Apixaban for Reduction in Stroke and Other Thromboembolic Events in Atrial Fibrillation trial (ARISTOTLE) for apixaban [18]. Moreover, recent important studies on DOACs suggest new possibilities in cardioversion and deserve to be examined [13,14,19]. These important pieces of evidence have been considered by the American Heart Association (AHA), American College of Cardiology (ACC), and the Heart Rhythm Society (HRS) in the recent 2019 Focused Update of the 2014 AF Guideline [13]. Thus, the aim of this review was to summarize the state-of-the-art methods regarding the use of DOACs in relation to cardioversion. 

## 2. DOACs for Cardioversion in Atrial Fibrillation

From a purely academic and explanatory point of view, we consider four different scenarios regarding cardioversion, which we examine point by point:

- Cardioversion of AF patient treated for >3 weeks with DOACs: we consider the subgroup analyses from RE-LY (dabigatran), ROCKET-AF (rivaroxaban), and ARISTOTLE (apixaban), including important news from the “eXplore the efficacy and safety of once-daily oral riVaroxaban for the prevention of caRdiovascular events in patients with nonvalvular aTrial fibrillation scheduled for cardioversion trial” (X-VeRT study) [20].
- Cardioversion of AF of >48 h in a patient not on DOACs: we consider the X-VeRT study [20] and “Edoxaban versus enoxaparin–warfarin in patients undergoing cardioversion of atrial fi brillation trial” (ENSURE AF) [21].
- Cardioversion of recent onset AF in an anticoagulation-naive patient: in this scenario, the results of the “Eliquis evaluated in acute cardioversion coMpared to usuAl treatmeNts for AnticoagulaTion in subjects with atrial fibrillation trial” (EMANATE trial) are very important [22].
- Patients with evidence of left atrial appendage (LAA) thrombus: we consider the few studies available in the literature on this scenario.

### 2.1. Cardioversion of AF Patient Treated for >3 Weeks with DOACs

The initial data on the use of DOACs in a clinical setting for cardioversion came from the post-hoc subgroup analysis of randomized control trials (RCTs) RE-LY [16], ROCKET AF [17], and ARISTOTLE [18]. 

In the RE-LY trial [16,23,24], from a total of 1983 cardioversions, patients received dabigatran 110 mg BID, dabigatran 150 mg BID, and warfarin. It was recommended that patients assigned to dabigatran receive at least 3 weeks of therapy before cardioversion. Stroke and systemic embolism rates at 30 days were 0.77 for dabigatran 110 mg BID, 0.60 for warfarin, 0.30 for dabigatran 150 mg BID, without significant differences among the treatment groups (dabigatran 110 mg versus warfarin, *p* = 0.71; dabigatran 150 mg versus warfarin, *p* = 0.40). Stroke and systemic embolism rates were similar in patients undergoing TEE before cardioversion (25% of patients assigned to dabigatran and 13% of patients assigned to warfarin) and in patients not performing TEE. Major bleeding rates were 1.7% for dabigatran 110 mg group, 0.6% for dabigatran 150 mg, and 0.6% for warfarin (dabigatran 110 mg versus warfarin, *p* = 0.06; dabigatran 150 mg versus warfarin, *p* = 0.99). 

The ROCKET AF post-hoc analysis [25] investigated patient outcomes with both cardioversion and catheter ablation procedures; 143 patients underwent electrical cardioversion, 142 underwent pharmacological cardioversion, and 79 underwent catheter ablation. The incidence of stroke or systemic embolism (1.88% versus 1.86%) and death (1.88% versus 3.73%) were similar in the rivaroxaban-treated and warfarin-treated groups. No data were available regarding the use of TEE pre-cardioversion. Major bleeding rates were 18.75% in the rivaroxaban group and 13.04% in the warfarin group. It is important to consider and remember that elective cardioversions were excluded by the enrolling protocol in ROCKET AF, i.e., patients who underwent cardioversion or ablation due to the fact of hemodynamic instability, progressive heart failure, or refractory symptoms despite optimal medical therapy [25].

In the ARISTOTLE trial [18,26], we found 540 cardioversions, and 265 patients received apixaban and 275 received warfarin. A TEE pre-procedural was performed in about 27% of cases. In the first 30 days after cardioversion, no patients experienced a thromboembolic event; one myocardial infarction (MI) and one major bleeding (MB) event occurred in each group, with two deaths in each group. In most cases, cardioversion occurred after months of treatment, with a mean time from enrolment to cardioversion of 243 ± 231 days for patients assigned to warfarin and of 251 ± 248 days for patients assigned to apixaban, far longer than the 3 weeks recommended by international guidelines [27].

Subgroup analyses from RE-LY, ROCKET-AF, and ARISTOTLE underline that electric cardioversion in patients treated with DOACs had a low and similar thromboembolic risk than patients treated with warfarin. According to these data, cardioversion without TEE seems reasonably safe under regular and continued DOAC intake.

### 2.2. Cardioversion of AF of >48 h in a Patient Not on DOACs Therapy

**Figure 2:**
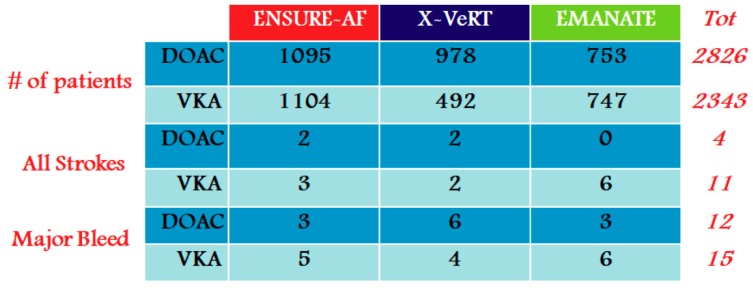
Summary of X-VeRT, ENSURE-AF, and EMANATE trials results.

The X-VeRT, ENSURE-AF, and EMANATE studies [20,21,22] were evaluated in the context of DOAC-naïve patients, respectively, with rivaroxaban (57% of patients), edoxaban (47%), and apixaban (61%) (see Figure 2). 

The X-VeRT study [20] is the first prospective randomized trial of DOACs in patients with atrial fibrillation undergoing elective cardioversion. Rivaroxaban was compared with dose adjusted VKA in the prevention of cardiovascular events in 1504 patients with non valvular atrial fibrillation (NVAF) scheduled for early or delayed cardioversion at the discretion of the local cardiologist investigator. In the early approach, oral anticoagulant therapy was given 1–5 days before cardioversion and, in the rivaroxaban arm, a cardioversion was performed at least 4 h after the first dose. In the delayed cardioversion approach, patients were anticoagulated for a range of 3–8 weeks before the procedure. Prophylaxis with rivaroxaban was considered adequate if the pill count was ≥80% in the three weeks preceding the cardioversion. The procedure was TEE guided in 65% of patients treated with an early strategy, whereas a TEE-guided cardioversion was performed in 10% of patients using a delayed strategy, with no significant difference in the rate of TEE employment among the two treatment groups. Primary efficacy endpoints were a composite of stroke and transient ischemic attack (TIA), non- systemic embolism, MI, CV death, while primary safety endpoints were MB. The primary efficacy endpoint occurred in 0.51% of patients in the rivaroxaban arm and 1.02% of patients in the VKAs arm (risk ratio 0.50; 95% CI 0.15–1.73). Majour bleeding (MB) occurred in 0.6% of patients in the rivaroxaban group and in 0.8% of patients in the VKAs group (risk ratio 0.76; 95% CI 0.21–2.67). Rivaroxaban was associated with low rates of adverse outcomes similar to those of VKAs even when data from the early and delayed strategies were analyzed separately. An important difference was found among the two strategies in terms of median time to cardioversion. This data were similar in the early strategy but significantly shorter in the delayed strategy using rivaroxaban versus warfarin (22 days rivaroxaban arm versus 30 days in VKA arm). Only 36% of patients anticoagulated with VKA were cardioverted as scheduled as the INR was not in range, in comparison with 77% in the rivaroxaban arm. According to data from X-VeRT, rivaroxaban can be considered as an effective and safe alternative to VKAs in patients with AF addressed to cardioversion, irrespective of the timing of the procedure. Moreover, X-VeRT showed that rivaroxaban may overcome critical limitations of VKA treatment in the setting of cardioversion, including a significant reduction in time to cardioversion and a considerable reduction of economic expenditure.

In the ENGAGE AF-TIMI 48 trial [28] few patients underwent electrical cardioversion and, therefore, we have limited data about edoxaban in the setting of procedures. Further data about anticoagulation with edoxaban in patients undergoing cardioversion are provided by the ENSURE-AF [21] which is the largest prospective randomized clinical trial of anticoagulation for cardioversion of patients with non-valvular atrial fibrillation. In this trial, 2199 patients scheduled for cardioversion were randomized (1:1) to edoxaban or enoxaparin/warfarin. As in the X-VeRT trial, patients were stratified in two different approaches. In the TEE-guided group, the TEE and cardioversion had to be executed within 3 days of randomization and patients randomly addressed to the edoxaban group had to begin treatment at least 2 h before electrical cardioversion. In the non-TEE-guided group, electrical cardioversion was executed at a minimum of 21 days following the start of anticoagulation. The primary endpoint (PE) was a composite of stroke, systemic embolic event (SEE), MI, and cardiovascular (CV) mortality, while the primary safety endpoint was a composite of major and non-major but clinically relevant bleeding (CRNM). The primary endpoint was similar with edoxaban compared to enoxaparin/warfarin in patients undergoing electrical cardioversion of NVAF (<1% edoxaban arm versus 1% enoxaparin/warfarin, odds ratio (OR) 0·46, 95% CI 0·12–1·43) and was independent of TE or previous anticoagulant therapy. The composite of the PE was numerically lower for edoxaban versus enoxaparin/warfarin and the main difference was due to the cardiovascular mortality, (0.1% in the edoxaban group versus 0.5% in the enoxaparin–warfarin). The primary safety endpoint occurred in 1.5% and 1.0% of patients in the edoxaban versus the enoxaparin/warfarin arm, respectively, and the results were statistically non-significant. There were numerically more major bleedings in the enoxaparin/warfarin arm, while more CRNM bleedings were found in the edoxaban arm. The net clinical outcome (composite of stroke/systemic embolism/myocardial infarction/cardiovascular death/major bleeding) was numerically lower in the edoxaban arm versus the enoxaparin/warfarin arm, but the result was statistically non-significant. In contrast with the X-VeRT study, there was no difference in time to delayed cardioversion among the two treatment groups. This probably means that the ENSURE-AF trial compared edoxaban with the optimized standard care of enoxaparin, bridging the pending therapeutic warfarin. The results suggest that edoxaban may be an effective and safe alternative to enoxaparin/VKA strategy and may allow prompt cardioversion to be performed when following a TEE-guided approach (edoxaban almost 2 h before ECV). 

### 2.3. Cardioversion of Recent Onset AF, in an Anticoagulation-Naive Patient

In this very important scenario, a key role is represented by EMANATE study [22]; this was the first study in anticoagulation-naive patients scheduled for cardioversion. All patients received <48 h anticoagulation and 61% were not anticoagulated prior to randomization. One thousand and thirty-eight patients underwent cardioversion, whereas 300 spontaneously restored sinus rhythm; 162 patients were not cardioverted; and in only 855 patients was imaging test (TEE or CT) performed. In some patients randomized to apixaban, according to the investigator, before the cardioversion, a single 10 mg loading dose of apixaban could be administered to achieve exposure at 2 h similar to steady state. Instead the maintenance dose was down titrated to 5 mg. Cardioversion could be performed 2 h after administration of the loading dose; 342 of patients received a loading dose of apixaban. The result of apixaban versus heparin/VKA group showed in EMANATE trial was: 0 versus 6 strokes (*p* = 0.0164), 3 versus 6 major bleeds, 2 versus 1 deaths, and no systemic embolic events in both groups. Among 342 patients receiving the loading dose of apixaban, there were 0 strokes, 1 major bleed, and 1 death. Finally, imaging identified left atrial appendage thrombi in 61 patients; all continued anticoagulation. Among those who underwent second imaging examination (37 ± 11 days after the initial imaging) thrombi resolved in 52% versus 56% in the apixaban and heparin/VKA groups. The EMANATE study supports the use of apixaban in patients with AF undergoing cardioversion. The novelty of this trial was the exclusive enrolment of anticoagulant-naïve patients (62% not receiving, 38% < 48 h) with recently detected AF (new onset or first diagnosed) with a focus on enrolling those amenable to early cardioversion. Also unique to the study, if an immediate cardioversion was planned, there was administration of a loading dose (10 mg) of apixaban at least 2 h before cardioversion. For this reason, potential participants were actively identified in hospital emergency departments which encouraged but did not mandate imaging (TEE or CT).

### 2.4. Management of a Patient with Documented Left Atrial Appendage Thrombus

The left atrial appendage is the site with the highest blood stasis which causes thrombus formation during atrial fibrillation [29]. In fact, about 90% of intracardiac thrombi in patients with cardioembolic events originally develop in the LAA. In patients with evidence of LAA thrombus cardioversion should not be performed. In patients with AF VKA, current guidelines recommend therapy for 3 weeks after diagnosis of an LA/LAA thrombus and long-term therapy for those with a documented residual thrombus [12,30]. Although relevant studies showing differences between DOAC and VKA (small data, although very interesting and encouraging, can be derived from the EMANATE study) do not yet exist, in the past few years, there some evidence has emerged supporting the use of DOACs for LA/LAA thrombus resolution, even if the data are limited to case studies or small case series [31]. The X-TRA study was the first prospective, multicenter study examining thrombus resolution with rivaroxaban in VKA-naïve patients or patients receiving suboptimal or ineffective VKA therapy [31]. This study showed that resolution or reduction of thrombus after rivaroxaban treatment was comparable to the results obtained with VKA therapy according to prior retrospective observational case series and the retrospective CLOT-AF registry [31]. The results suggest that rivaroxaban seems to be a potential option for the treatment of TEE-detected LA/LAA thrombi in patients with AF [31]. Even dabigatran has generated encouraging data regarding its use for LA/LAA thrombus resolution [32]. In a small study with a total of 58 AF patients with LAA thrombus, Xiao et al. [32] demonstrated that dabigatran was effective in the dissolution of LAA thrombus in patients with AF. Ongoing trial RE-LATED-AF will make further clarifications about the use of DOACs in this complex and poorly studied scenario [33]. 

## 3. Conclusions

For several years, warfarin has been the primary oral anticoagulant used for patients with AF [34], affirming its superiority over acetylsalicylic acid in reducing thromboembolic risk [1,11,12,13]. Since 2009 until 2013, the four DOAC registered RCTs have paved the way for a more optimal prevention of thromboembolic risk in patient with AF, reducing hemorrhagic risk; in particular, all DOACs significantly reduced the risk of intracranial hemorrhage (ICH), principally in more complex categories of patients such as in the elderly, frail, and patients with comorbidities (Figure 2) [5,6,35]. 

Direct oral anticoagulants (DOACs) offer several potential advantages over warfarin therapy in the setting of cardioversion, including removing the need for routine laboratory monitoring and heparin bridging therapy (the latter of which is very important, especially in the case of surgical interventions), as well as having predictable pharmacokinetic and security pharmacodynamic profiles. 

Finally, DOACs demonstrated good predictable onset of anticoagulation (since 1 to maximum 4 h) compared with 48 to 72 h for warfarin and up to 5 to 7 days to reach steady state [36,37]. In a very interesting random-effects meta-analysis performed by Brunetti et al. [38], a total of 8564 patients undergoing both electrical and pharmacologic cardioversions for NVAF were included observing, one more time, the effectiveness and safety of DOACs in patients undergoing NVAF.

Unfortunately, today, head-to-head studies do not exist and direct comparisons between DOACs are not possible. Even though other studies concerning DOACs in the context of AF are ongoing (for example, the RE-LATED AF-AFNET 7 trial [33]) and further real-life data are needed, DOACs therapy is an effective and safe strategy in the context of atrial fibrillation patients scheduled for cardioversion.

## References

[1] Steffel J., Verhamme P., Potpara T.S., Albaladejo P., Antz M., Desteghe L., Georg Haeusler K., Oldgren J., Reinecke H., Roldan-Schilling V. (2018). ESC Scientific Document Group. The 2018 European Heart Rhythm Association. Practical Guide on the use of non-vitamin K antagonist oral anticoagulants in patients with atrial fibrillation. Eur. Heart J..

[2] Rietbrock S., Heeley E., Plumb J., van Sta T. (2008). Chronic atrial fibrillation: Incidence, prevalence, and prediction of stroke using the Congestive heart failure, Hypertension, Age >75, Diabetes mellitus, and prior Stroke or transient ischemic attack (CHADS2) risk stratification scheme. Am. Heart J..

[3] Kulbertus H., Lancellotti P. (2014). Fibrillation, an epidemic in the elderly?. Rev. Med. Liege.

[4] Puccio D., Novo G., Baiamonte V., Nuccio A., Fazio G., Corrado E., Coppola G., Muratori I., Vernuccio L., Novo S. (2009). Atrial fibrillation and mild cognitive impairment: What correlation?. Minerva Cardioangiol..

[5] Manno G., Novo G., Corrado E., Coppola G., Novo S. (2019). Use of direct oral anticoagulants in very elderly patients: A case report of apixaban in an ultracentenary patient. J. Cardiovasc. Med. (Hagerstown).

[6] Russo V., Carbone A., Rago A., Golino P., Nigro G. (2019). Direct Oral Anticoagulants in octogenarians with atrial fibrillation: it’s never too late. J. Cardiovasc. Pharmacol..

[7] McNamara R.L., Tamariz L.J., Segal J.B., Bass E.B. (2003). Management of atrial fibrillation: Review of the evidence for the role of pharmacologic therapy, electrical cardioversion, and echocardiography. Ann. Intern. Med..

[8] Naccarelli G.V., Dell’Orfano J.T., Wolbrette D.L., Patel H.M., Luck J.C. (2000). Cost-effective management of acute atrial fibrillation: Role of rate control, spontaneous conversion, medical and direct current cardioversion, transesophageal echocardiography, and antiembolic therapy. Am. J. Cardiol..

[9] Di Fusco S.A., Colivicchi F., Aspromonte N., Tubaro M., Aiello A., Santini M. (2017). Direct oral anticoagulants in patients undergoing cardioversion: Insight from randomized clinical trials. Monaldi Arch. Chest Dis..

[10] Russo V., Bottino R., Rago A., Micco P.D., D’Onofrio A., Liccardo B., Golino P., Nigro G. (2019). Atrial Fibrillation and Malignancy: The Clinical Performance of Non-Vitamin K Oral Anticoagulants-A Systematic Review. Semin. Thromb. Hemost..

[11] Andò G., Trio O. (2016). New oral anticoagulants versus Warfarin in patients undergoing cardioversion of atrial fibrillation. Int. J. Cardiol..

[12] Andò G., Trio O., Carerj S. (2015). New oral anticoagulants versus vitamin K antagonists before cardioversion of atrial fibrillation: A meta-analysis of data from 4 randomized trials. Expert Rev. Cardiovasc. Ther..

[13] Kirchhof P., Benussi S., Kotecha D., Ahlsson A., Atar D., Casadei B., Castella M., Diener H.C., Heidbuchel H., Hendriks J. (2016). ESC Scientific Document Group. 2016 ESC Guidelines for the management of atrial fibrillation developed in collaboration with EACTS. Eur. Heart J..

[14] January C.T., Wann L.S., Calkins H., Chen L.Y., Cigarroa J.E., Cleveland J.C., Ellinor P.T., Ezekowitz M.D., Field M.E., Furie K.L. (2019). 2019 AHA/ACC/HRS Focused Update of the 2014 AHA/ACC/HRS Guideline for the Management of Patients With Atrial Fibrillation: A Report of the American College of Cardiology/American Heart Association Task Force on Clinical Practice Guidelines and the Heart Rhythm Society. J. Am. Coll. Cardiol..

[15] Trujillo T.C., Dobesh P.P., Crossley G.H., Finks S.W. (2019). Contemporary Management of Direct Oral Anticoagulants During Cardioversion and Ablation for Nonvalvular Atrial Fibrillation. Pharmacotherapy.

[16] Connolly S.J., Ezekowitz M.D., Yusuf S., Eikelboom J., Oldgren J., Parekh A., Pogue J., Reilly P.A., Themeles E., Varrone J. (2009). for the RE-LY Steering Committee and Investigators. Dabigatran versus warfarin in patients with atrial fibrillation. N. Engl. J. Med..

[17] Patel M.R., Mahaffey K.W., Garg J., Pan G., Singer D.E., Hacke W., Breithardt G., Halperin J.L., Hankey G.J., Piccini J.P. (2011). ROCKET AF Investigators. Rivaroxaban versus warfarin in nonvalvular atrial fibrillation. N. Engl. J. Med..

[18] Granger C.B., Alexander J.H., McMurray J.J., Lopes R.D., Hylek E.M., Hanna M., Al-Khalidi H.R., Ansell J., Atar D., Avezum A. (2011). Apixaban versus warfarin in patients with atrial fibrillation. N. Engl. J. Med..

[19] Reilly P.A., Lehr T., Haertter S., Connolly S.J., Yusuf S., Eikelboom J.W., Ezekowitz M.D., Nehmiz G., Wang S., Wallentin L. (2014). The effect of dabigatran plasma concentrations and patient characteristics on the frequency of ischemic stroke and major bleeding in atrial fibrillation patients: The RE-LY Trial (Randomized Evaluation of Long-Term Anticoagulation Therapy). J. Am. Coll. Cardiol..

[20] Cappato R., Ezekowitz M.D., Klein A.L., Camm A.J., Ma C.S., Le Heuzey J.Y., Talajic M., Scanavacca M., Vardas P.E., Kirchhof P. (2014). X-VeRT Investigators. Rivaroxaban vs. vitamin K antagonists for cardioversion in atrial fibrillation. Eur. Heart J..

[21] Goette A., Merino J.L., Ezekowitz M.D., Zamoryakhin D., Melino M., Jin J., Mercuri M.F., Grosso M.A., Fernandez V., Al-Saady N. (2016). ENSURE-A investigators. Edoxaban versus enoxaparin-warfarin in patients undergoing cardioversion of atrial fibrillation (ENSURE-AF): A randomised, open-label, phase 3b trial. Lancet.

[22] Ezekowitz M.D., Pollack C.V., Halperin J.L., England R.D., VanPelt Nguyen S., Spahr J., Sudworth M., Cater N.B., Breazna A., Oldgren J. (2018). Apixaban compared to heparin/vitamin K antagonist in patients with atrial fibrillation scheduled for cardioversion: The EMANATE trial. Eur. Heart J..

[23] Lin H.D., Lai C.L., Dong Y.H., Tu Y.K., Chan K.A., Suissa S. (2019). Re-evaluating Safety and Effectiveness of Dabigatran Versus Warfarin in a Nationwide Data Environment: A Prevalent New-User Design Study. Drugs Real World Outcomes.

[24] Nagarakanti R., Ezekowitz M.D., Oldgren J., Yang S., Chernick M., Aikens T.H., Flaker G., Brugada J., Kamensky G., Parekh A. (2011). Dabigatran versus warfarin in patients with an analysis of patients undergoing cardioversion. Circulation.

[25] Piccini J.P., Stevens S.R., Lokhnygina Y., Patel M.R., Halperin J.L., Singer D.E., Hankey G.J., Hacke W., Becker R.C., Nessel C.C. (2013). Outcomes after cardioversion and atrial fibrillation ablation in patients treated with rivaroxaban and warfarin in the ROCKET AF trial. J. Am. Coll. Cardiol..

[26] Lip G.Y.H., Khan A.A., Olshansky B. (2019). Short-Term Outcomes of Apixaban Versus Warfarin in Patients With Atrial Fibrillation. Circulation.

[27] Flaker G., Lopes R.D., Al-Khatib S.M., Hermosillo A.G., Hohnloser S.H., Tinga B., Zhu J., Mohan P., Garcia D., Bartunek J. (2014). Efficacy and safety of apixaban in patients after cardioversion for atrial fibrillation. J. Am. Coll. Cardiol..

[28] Ruff C.T., Giugliano R.P., Braunwald E., Morrow D.A., Murphy S.A., Kuder J.F., Deenadayalu N., Jarolim P., Betcher J., Shi M. (2015). Association between edoxaban dose, concentration, anti-Factor Xa activity, and outcomes: An analysis of data from the randomised, double-blind ENGAGE AF-TIMI 48 trial. Lancet.

[29] Masci A., Barone L., Dedè L., Fedele M., Tomasi C., Quarteroni A., Corsi C. (2019). The Impact of Left Atrium Appendage Morphology on Stroke Risk Assessment in AtrialFibrillation: A Computational Fluid Dynamics Study. Front. Physiol..

[30] Stabile G., Russo V., Rapacciuolo A., De Divitiis M., De Simone A., Solimene F., D’Onofrio A., Iuliano A., Maresca G., Esposito F. (2015). Transesophageal echocardiograpy in patients with persistent atrial fibrillation undergoing electrical cardioversion on new oral anticoagulants: A multi center registry. Int. J. Cardiol..

[31] Lip G.Y., Hammerstingl C., Marin F., Cappato R., Meng I.L., Kirsch B., van Eickels M., Cohen A. (2016). X-TRA study and CLOT-AF registry investigators. Left atrial thrombus resolution in atrial fibrillation or flutter: Results of a prospective study with rivaroxaban (X-TRA) and a retrospective observational registry providing baseline data (CLOT-AF). Am. Heart J..

[32] Xing X.F., Liu N.N., Han Y.L., Zhou W.W., Liang M., Wang Z.L. (2018). Anticoagulation efficacy of dabigatran etexilate for left atrial appendage thrombus in patients with atrial fibrillation by transthoracic and transesophageal echocardiography. Medicine (Baltimore).

[33] Ferner M., Wachtlin D., Konrad T., Deuster O., Meinertz T., von Bardeleben S., Münzel T., Seibert-Grafe M., Breithardt G., Rostock T. (2016). Rationale and design of the RE-LATED AF-AFNET 7 trial: Resolution of Left atrial-Appendage Thrombus-Effects of Dabigatran in patients with Atrial Fibrillation. Clin. Res. Cardiol..

[34] European Atrial Fibrillation Trial Study (1995). Optimal oral anticoagulant therapy in patients with non rheumatic atrial fibrillation and recent cerebral ischemia. N. Engl. J. Med..

[35] Rago A., Papa A.A., Cassese A., Arena G., Magliocca M.C.G., D’Onofrio A., Golino P., Nigro G., Russo V. (2019). Clinical Performance of Apixaban vs. Vitamin K Antagonists in Patients with Atrial Fibrillation Undergoing Direct Electrical Current Cardioversion: A Prospective Propensity Score-Matched Cohort Study. Am. J. Cardiovasc. Drugs.

[36] Russo V., Rago A., Proietti R., Di Meo F., Papa A., Calabrò P., D’Onofrio A., Nigro G., AlTurki A. (2017). Efficacy and safety of the target specific oral anticoagulants for stroke prevention in atrial fibrillation: The real-life evidence. Ther. Adv. Drug Saf..

[37] Gibson C.M., Basto A.N., Howard M.L. (2018). Direct Oral Anticoagulants in Cardioversion: A Review of Current Evidence. Ann. Pharmacother..

[38] Brunetti N.D., Tarantino N., De Gennaro L., Correale M., Santoro F., Di Biase M. (2018). Direct oral anti-coagulants compared to vitamin-K antagonists in cardioversion of atrial fibrillation: An updated meta-analysis. J. Thromb. Thromb..

